# Hyperglycaemia-induced diabetic amyotrophy: a case report from a family medicine clinic

**DOI:** 10.3399/bjgpopen20X101026

**Published:** 2020-02-05

**Authors:** Apichai Wattanapisit, Sanhapan Wattanapisit, Jaruporn Thongruch

**Affiliations:** 1 Associate Professor of Family Medicine, School of Medicine, Walailak University, Nakhon Si Thammarat, Thailand; 2 Family Physician, Family Medicine Clinic, Walailak University Hospital, Nakhon Si Thammarat, Thailand; 3 Family Physician, Family Medicine Unit, Thasala Hospital, Nakhon Si Thammarat, Thailand; 4 Registered Nurse, Family Medicine Clinic, Walailak University Hospital, Nakhon Si Thammarat, Thailand

**Keywords:** diabetes, type 2 diabetes mellitus, hyperglycaemia, family medicine, primary health care

## Introduction

Type two diabetes mellitus (T2DM) is a common health problem in primary care. Hyperglycaemia is a manifestation of T2DM and causes several complications. Diabetic neuropathies are a common complication and have been found in several forms and presentations.^1^ This article reports on a patient with T2DM, who presented in primary care with progressive lower extremity weakness and walking difficulty.

## Case report

A 64-year-old male visited a family medicine clinic due to lower extremity weakness lasting 1 month. The patient could not walk without assistance or walking aids. He was fully conscious and presented at the clinic in a wheelchair. He had numbness and a tingling sensation in his feet. There had been a 7 kg weight loss (from 60 to 53 kg) in a month. The patient had a past medical history of T2DM, diagnosed approximately 10 years previously. His medications were metformin 1000 mg daily and glipizide 10 mg daily; though he had irregularly attended his diabetes clinics, his adherence to medications was variable and he had had no recent follow-up or blood tests.

His vital signs were blood pressure, 141/93 mmHg; pulse, 108 beats per minute; respiratory rate, 20 breaths per minute; and temperature, 36.5°C. Physical examination of the nervous system revealed a decrease in motor power related to both hip flexion and elbow extension (grade 4). Pain sensation (tested by a pin prick test) was normal. Deep tendon reflexes of the knees and ankles indicated a slightly slow response (1+). The rest of his physical examination was normal.

Blood tests indicated a fasting plasma glucose (FPG) level of 650 mg/dL (36.1 mmol/L). Serum electrolytes were as follows: sodium, 135.3 mEq/L, potassium, 4.7 mEq/L, chloride, 97.3 mEq/L; and bicarbonate, 22.4 mEq/L. Serum creatinine was 1.4 mg/dL, and estimated glomerular filtration rate was 48.8 ml/min/1.73 m^2^. Thyroid function tests were normal. A glycated haemoglobin (HbA_1c_) was not taken at initial presentation.

The patient was referred to a district hospital because of symptomatic hyperglycaemia and poor mobility. During the patient’s 4-day hospital stay, the injectable insulin was prescribed to control his glucose level. The plasma glucose level was under control during the hospital stay and the weakness of the lower extremities improved. The patient could walk without assistance after hospital discharge. Subcutaneous insulin (30%/isophane 70% [mixtard 100 IU/ml] at 26 IU before breakfast and 14 IU before dinner) was prescribed after discharge from hospital. Six weeks later, the patient could walk to attend a follow-up visit at the family medicine clinic. Physical examination of the nervous system revealed normal muscle strength and normal muscle tone. The patient gained 5.9 kg of body weight (58.9 kg). The FPG was 281 mg/dL (15.6 mmol/L), and HbA_1c_ was 10.9%. By the next follow-up visit (4 weeks later), the patient had made a full recovery from lower extremity weakness and could perform all activities of daily living. His body weight was 63 kg. His glycaemic test results were improved (FPG, 121 mg/dL [6.7 mmol/L]; HbA_1c_, 6.6%).

## Discussion

The patient was diagnosed with a form of diabetic neuropathy. The symptoms were reversed after using insulin to treat hyperglycaemia. The most likely diagnosis was diabetic amyotrophy (Burns–Garland syndrome). This syndrome was first described in 1890 by Burns, and Garland defined the term diabetic amyotrophy in 1955.^2,3^ Diabetic amyotrophy has been found in less than 1% of patients with diabetes mellitus, and affects males more frequently than females.^1,3^ The diagnosis of diabetic amyotrophy can be made clinically.^3^ Clinical features include neuropathic pain affecting the lower back or lower extremities, weakness, and weight loss.^1^ Typically, diabetic amyotrophy begins with pain in the buttock or one thigh, expanding to the other parts of the same leg, and then the opposite leg due to the involvement of the lumbosacral roots, plexus, and peripheral nerves.^3^ Muscle weakness will present after a few weeks of the onset of neuropathic pain.^3^ Subsequently, muscle wasting is a cause of weight loss.^3^ By the time the patient seeks medical consultation, the symptoms may involve both lower extremities.^3^ Investigations (such as electromyography, nerve conduction study, cerebrospinal fluid examination, nerve biopsy, and imaging) may be helpful to exclude other aetiologies.^1,3^ The most likely cause of diabetic amyotrophy is microvasculitis, but metabolic causes (such as hyperglycaemia) may be responsible for the pathophysiology.^3,4^

The patient was treated with insulin to lower plasma glucose. Subsequently, the symptoms of weakness dramatically improved, and the tingling sensation stopped. FPG and HbA_1c_ steadily improved over a 10-week treatment course. The patient’s body weight increased to its previous level. In general, supportive management, including maintaining good diabetic control, pain control, and physiotherapy, are the main treatments.^3^ A short course of corticosteroids may be effective for pain control in diabetic amyotrophy.^3^ Based on the immune-mediated microvasculitis pathogenesis, intravenous immunoglobulin has been used as a treatment for diabetic amyotrophy.^4,5^ However, a Cochrane review reports that there is insufficient evidence to support the effects of immunotherapy in the treatment of diabetic amyotrophy.^4^

Hyperglycaemia is the most likely cause of the diabetic amyotrophy in this patient. The symptoms of diabetic amyotrophy can last for many months.^3^ For this patient, if the symptoms were prolonged after glycaemic control, other differential diagnoses would have been investigated. In addition to the diagnosis of this rare condition and its management, the patient had poorly controlled diabetes mellitus. Understanding the psychosocial needs of patients is crucial to overcoming barriers associated with self-care and treatment adherence.^6^

Physicians in primary care settings should be aware of the variety of presentations of diabetic neuropathies. Generalised and focal or multifocal neuropathies can be explained by different aetiologies and pathogenesises; for example, diabetic amyotrophy is a presentation of lumbosacral radiculoplexus neuropathy.^1^ Diabetic amyotrophy is a rare complication of T2DM. Therefore, before making a diagnosis of diabetic amyotrophy, primary care physicians should rule out serious medical conditions requiring emergency care. History-taking and physical examination are essential to help differentiate diabetic amyotrophy from other neurological disorders.
